# Oxiconazole Potentiates Gentamicin against Gentamicin-Resistant Staphylococcus aureus *In Vitro* and *In Vivo*

**DOI:** 10.1128/spectrum.05031-22

**Published:** 2023-07-10

**Authors:** Grace Kaul, Abdul Akhir, Manjulika Shukla, Hasham Shafi, Ravikumar Akunuri, Gaurav Pawar, Mahammad Ghouse, Nanduri Srinivas, Sidharth Chopra

**Affiliations:** a Division of Molecular Microbiology and Immunology, Lucknow, Uttar Pradesh, India; b Division of Pharmaceutics & Pharmacokinetics, CSIR-Central Drug Research Institute, Lucknow, Uttar Pradesh, India; c Academy of Scientific and Innovative Research (AcSIR), Ghaziabad, India; d Department of Chemical Sciences, NIPER Hyderabad, Hyderabad, Telengana, India; University of Warwick

**Keywords:** oxiconazole, *Staphylococcus aureus*, drug resistance, drug repurposing, multidrug resistance

## Abstract

Amid the mounting burden of multidrug-resistant (MDR) bacterial infections on health care worldwide, drug repurposing, a time and cost-effective strategy to identify new applications for drugs approved for other indications, can effectively fill the void in the current antibiotic pipeline. In this study, we have repurposed a topical antifungal agent, oxiconazole, in combination with gentamicin against skin infections caused by multidrug-resistant Staphylococcus aureus. Oxiconazole was identified as having antibacterial activity against S. aureus via whole-cell screening assays against clinically relevant bacterial pathogens. It exhibited a potent *in vitro* profile, including equipotent activity against clinical drug-susceptible and -resistant S. aureus and *Enterococcus* spp. Checkerboard assays and time-kill kinetics studies demonstrated its concentration-dependent killing and ability to synergize with the approved antibiotics daptomycin and gentamicin against susceptible and MDR S. aureus strains. Oxiconazole also significantly eradicated preformed S. aureus biofilms *in vitro*. Eventually, in an assessment of its ability to generate resistant S. aureus mutants via serial passaging, oxiconazole displayed an extremely low propensity for developing stable resistance in S. aureus. Its *in vivo* efficacy alone and in combination with synergistic antibiotics was assessed in a murine superficial skin infection model of S. aureus, where it strongly synergized with gentamicin, exhibiting superior activity to the untreated control and drug-alone treatment groups. Thus, oxiconazole can be repurposed as an antibacterial alone and in combination with gentamicin against susceptible and gentamicin-resistant S. aureus infections.

**IMPORTANCE** Staphylococcus aureus, which causes the majority of nosocomial and community-acquired infections globally, is a WHO high-priority pathogen for antibiotic research and development. In addition to invasive infections, it is the causative agent of moderate to severe skin infections, with an increasing prevalence of infections caused by MDR strains such as methicillin-resistant S. aureus (MRSA). Our study highlights the repurposing of oxiconazole, a topical antifungal agent, as an ideal candidate for combination therapy with gentamicin against susceptible and drug-resistant S. aureus skin infections due to its extremely low propensity for resistance generation in S. aureus, activity against MDR strains, bactericidal killing kinetics alone and in combination, broad antifungal efficacy, and excellent safety and tolerability profile.

## INTRODUCTION

Antimicrobial resistance (AMR) is a silent pandemic posing a global health crisis in the 21st century (1, 2). Experts have noted that multidrug-resistant (MDR) infections could, annually, kill 10 million people worldwide by 2050, and by 2030, AMR could force up to 24 million people into extreme poverty (2). Medical procedures and treatment of common infections are at high risk due to AMR, exacerbated by the slow-moving rate of novel antibiotic discovery (3, 4).

Among the leading pathogens associated with resistance and classified as a high-priority pathogen by the WHO for antibiotic research and development is Staphylococcus aureus, whish causes the majority of nosocomial and community-acquired infections worldwide (1, 5). Other than invasive infections such as endocarditis, osteomyelitis, bacteremia, and hospital-acquired pneumonia, S. aureus is the causative agent of moderate to severe skin infections representing a considerable public health burden due to their high-frequency occurrences, especially in United States (6–8). The increasing burden of infections caused by MDR strains such as methicillin-resistant S. aureus (MRSA) combined with the paucity of efficient antibiotics has led to the urgent need to investigate antibacterials that can circumvent the existing resistance mechanisms and have low propensity for resistance generation among bacteria (9, 10).

Conventional *de novo* drug discovery is a lengthy, laborious process that is accompanied by hefty development expenses. In contrast, drug repurposing provides a more dynamic, cost-effective, and feasible option for swiftly replenishing the increasingly dwindling discovery pipeline (11). Its intent is to study novel applications for previously authorized pharmaceuticals to minimize the period between drug discovery and deployment, the expense of drug development, and the inherent risk of drug innovation (12). In addition, combination therapies with standard of care (SOC) antibiotics possess many advantages over monotherapy and aid in ameliorating the lacunae of the currently employed antibiotic regimens (13).

In this context, we discovered to oxiconazole exhibit significant antimicrobial activity against Staphylococcus aureus and *Enterococcus* spp. Since its introduction, many studies have reported oxiconazole and oxiconazole-like analogues with a broad spectrum of activity, including antifungal, antileishmanial, antibacterial, and anticancer properties (14–20). However, detailed antibacterial analysis of oxiconazole has not been previously conducted. In this study, we describe for the first time, to our knowledge, detailed antistaphylococcal activity of oxiconazole in combination with gentamicin *in vitro* and *in vivo* against drug-resistant S. aureus infections.

## RESULTS AND DISCUSSION

### Oxiconazole demonstrates potent antimicrobial activity against S. aureus and *Enterococcus* spp.

The screening of the Prestwick chemical library identified oxiconazole as a nonantibacterial molecule preferentially demonstrating antibacterial activity against the Gram-positive pathogens S. aureus and *Enterococcus* spp. but was inactive against Gram-negative bacterial (GNB) pathogens (Table 1). From a drug-repurposing perspective, oxiconazole offered an excellent safety and tolerability profile with a long history of clinical utilization for topical administration thus was advanced for detailed investigation.

**TABLE 1:** MIC of oxiconazole and comparator drugs against ESKAPE pathogen panel

Compound	MIC (mg/L) for:				
	S. aureus ATCC 29213	E. coli ATCC 25922	P. aeruginosa ATCC 27853	K. pneumoniae BAA-1705	A. baumannii BAA-1605
Oxiconazole	2	>64	>64	>64	>64
Levofloxacin	0.125	0.015	1	64	8

One of the primary reasons compounds do not exhibit activity against GNBs is a lack of entry through the outer membrane (OM), a well-known resistance mechanism (21). In order to investigate this further, we determined the MIC of oxiconazole against the GNBs Escherichia coli ATCC 25922 and Acinetobacter baumannii BAA-1605 in the presence of an OM-permeabilizing agent, polymyxin B nonapeptide (PMBN), at a subinhibitory concentration (10 mg/L), and the results are shown in Table 2. Interestingly, oxiconazole exhibited activity (MIC, 4 to 8 mg/L) against both pathogens that was comparable to its activity against S. aureus. These findings suggest that oxiconazole has broad-spectrum antibacterial efficacy but only in the presence of OM-permeabilizing agents, particularly against GNBs. The permeabilization of the OM of Gram-negative bacteria suggests a cytosolic target or targets, which may be conserved across bacterial species.

**TABLE 2:** MIC of oxiconazole in combination with PMBN (0.01 mg/L) against Gram-negative pathogens^*a*^

Bacterial strain	MIC (mg/L) for:								
	PMBN alone	Oxiconazole and PMBN		Rifampicin and PMBN		Vancomycin and PMBN		Levofloxacin and PMBN	
		(−)	(+)	(–)	(+)	(–)	(+)	(–)	(+)
Escherichia coli ATCC 25922	>64	>512	4	8	0.06	512	128	0.03	0.015
Acinetobacter baumannii BAA-1605	>64	>512	8	2	0.03	256	64	8	8

a PMBN, polymyxin B nonapeptide hydrochloride. −indicates absence and + indicates presence of PMBN.

### Oxiconazole expresses equipotent antimicrobial activity against clinical MDR S. aureus and *Enterococcus* sp. isolates.

Additionally, an extended MRSA, vancomycin-resistant S. aureus (VRSA), and *Enterococcus* strain panel comprising clinical isolates with clearly established and described resistance patterns was used to assess the antibacterial efficacy of oxiconazole. Oxiconazole was equally effective against drug-susceptible and drug-resistant strains of S. aureus and *Enterococcus*, as shown in Table 3, and there was no discernible difference in MIC with respect to virulence factors such as Panton-Valentine leucocidin or different types of *mec* cassettes. This suggests a novel mechanism of action and a lack of cross-resistance with other clinically used antibiotics.

**TABLE 3:** MIC of oxiconazole against clinical strains of drug-resistant S. aureus and *Enterococcus* spp.^*a*^

Designation	S. aureus strain		Antibiotic resistance to:	Molecular detail(s) of strains	MIC of oxiconazole (mg/L)
MSSA		ATCC 29213	None	Type strain	2
MRSA		NRS100	Meth, tetra	Contains subtype I *mec* cassette and large variety of virulence factors	4
		NRS119	Genta, line, meth, tri/sul	Contains subtype IV *mec* cassette and G2576T mutation in domain V in 23S rRNA genes	4
		NRS129	Chlor	*mecA* negative	4
		NRS186	Meth, levo, mero	USA 300 type CA-MRSA, PVL factor positive and contains *mec* type IV cassette	4
		NRS191	Meth, levo, mero	USA 600 type CA-MRSA, PVL factor negative and contains *mec* type II cassette	4
		NRS192	Meth, levo, mero, ery	CA-MRSA, PVL factor negative and contains *mec* type II cassette	4
		NRS193	Meth, levo, mero	CA-MRSA, PVL factor negative and contains *mec* type II cassette	4
		NRS194	Meth, mero	CA-MRSA, PVL factor positive and contains *mec* type V cassette	4
		NRS198	Meth, mero, levo	USA 100 type CA-MRSA, PVL factor negative and contains *mec* type II cassette	4
VRSA		VRS 1	Meth, levo, van, mero, genta, spec, teico	USA 100, contains *mec* subtype II cassette and *vanA*, negative for *vanB*, *vanC1*, *vanC2*, *vanD*, *vanE*, PVL, and ACME	4
		VRS 4	Meth, levo, van, mero, genta, spec, teico	USA 100, contains *mec* subtype II cassette and *vanA*, negative for *vanB*, *vanC1*, *vanC2*, *vanD*, *vanE*, PVL, and ACME	4
		VRS 12	Meth, levo, van, mero, genta, spec, teico	Data not available	4
*Enterococcus*		NR 31903	Van, amp	Genome-sequenced strain	4
		NR 31884	Genta	Hemolytic, cytolytic isolate; genome-sequenced strain	16
		NR 31885	Genta	Cytolytic isolate; genome-sequenced strain	8
		NR 31912	Van	Genome-sequenced strain; (GenBank accession no. AJDX00000000)	1

a PFGE, pulsed-field gel electrophoresis; PVL, Panton-Valentine leucocidin virulence factor; ACME, arginine catabolic mobile element; CA, community-acquired; meth, methicillin; mero, meropenem; tetra, tetracycline; van, vancomycin; teico, teicoplanin; genta, gentamicin; line, linezolid; chlor, chloramphenicol; tri/sul, trimeth/sulfa; ery, erythromycin; spec, spectinomycin; AMP, ampicillin; S, susceptible; R, resistant.

### Oxiconazole exhibits concentration-dependent bactericidal kinetics against S. aureus ATCC 29213.

**FIG 1:**
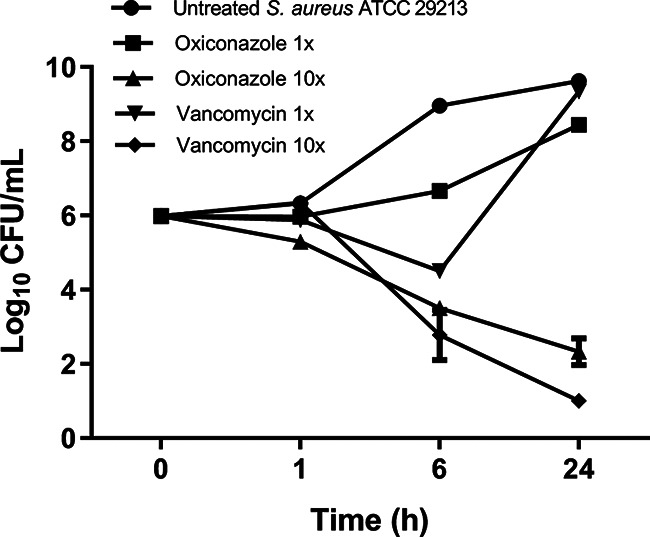
Time-kill kinetics of oxiconazole and comparator antibiotics against S. aureus ATCC 29213. The error bars denote the standard error of the mean (SEM) gained from duplicate samples used for each antibiotic/compound examined in two independent experiments (*n* = 8). Time-kill kinetics of oxiconazole alone with comparator antibiotics and in combination with gentamicin and daptomycin against S. aureus ATCC 29213 was performed in the same set of experiments, and results are shown separately in this figure and Fig. 2A and B for clarity. Log_10_ CFU/mL values for the untreated control (S. aureus ATCC 29213) and the oxiconazole (1× MIC) treatment group are unchanged in this figure and Fig. 2A and B.

In order to determine whether oxiconazole exhibits bactericidal or bacteriostatic activity, the killing kinetics of oxiconazole were assessed at 1× and 10× MIC with vancomycin as a control against S. aureus ATCC 29213. As depicted in Fig. 1, oxiconazole reduced the CFU/mL by ~7.29 log_10_, and vancomycin reduced the CFU/mL by ~8.6 log_10_ in 24 h at 10× MIC compared to the untreated control, with no sign of regrowth. Oxiconazole reduced the CFU/mL by ~1.2 log_10_ at 1× MIC in 24 h compared to the untreated control, while vancomycin at 1× MIC reached the same CFU/mL as the untreated control in 24 h. Taking these findings together, oxiconazole demonstrates concentration-dependent bactericidal kinetics that is comparable to vancomycin.

### Oxiconazole synergizes with gentamicin and daptomycin against S. aureus ATCC 29213.

The ability of oxiconazole to synergize with FDA-approved antibiotics used for the treatment of S. aureus was investigated, since combination therapy is a promising approach to battle growing AMR. Oxiconazole synergizes with daptomycin and gentamicin against S. aureus ATCC 29213 (fractional inhibitory concentration [ΣFIC], ≤0.5), as shown in Table 4, while not interacting with the other drugs tested.

**TABLE 4:** Interaction of oxiconazole with approved drugs against S. aureus ATCC 29213

Drug/compound	MIC (mg/L)	MIC (mg/L) of oxiconazole in presence of drug A	MIC (mg/L) of oxiconazole in presence of drug B	FIC (A)	FIC (B)	FIC (FIC A + FIC B)	Inference
Oxiconazole	4						
Ceftazidime	16	1	8	0.25	0.5	0.75	No interaction
Daptomycin	1	1	0.25	0.25	0.25	0.5	Synergistic
Gentamicin	0.5	1	0.125	0.25	0.25	0.5	Synergistic
Linezolid	4	4	4	1	1	2	No interaction
Levofloxacin	0.25	4	0.25	1	1	2	No interaction
Meropenem	0.125	2	0.0625	0.5	0.5	1	No interaction
Minocycline	0.5	2	0.25	0.5	0.5	1	No interaction
Rifampicin	0.0075	4	0.0075	1	1	2	No interaction
Vancomycin	1	4	1	1	1	2	No interaction

**FIG 2:**
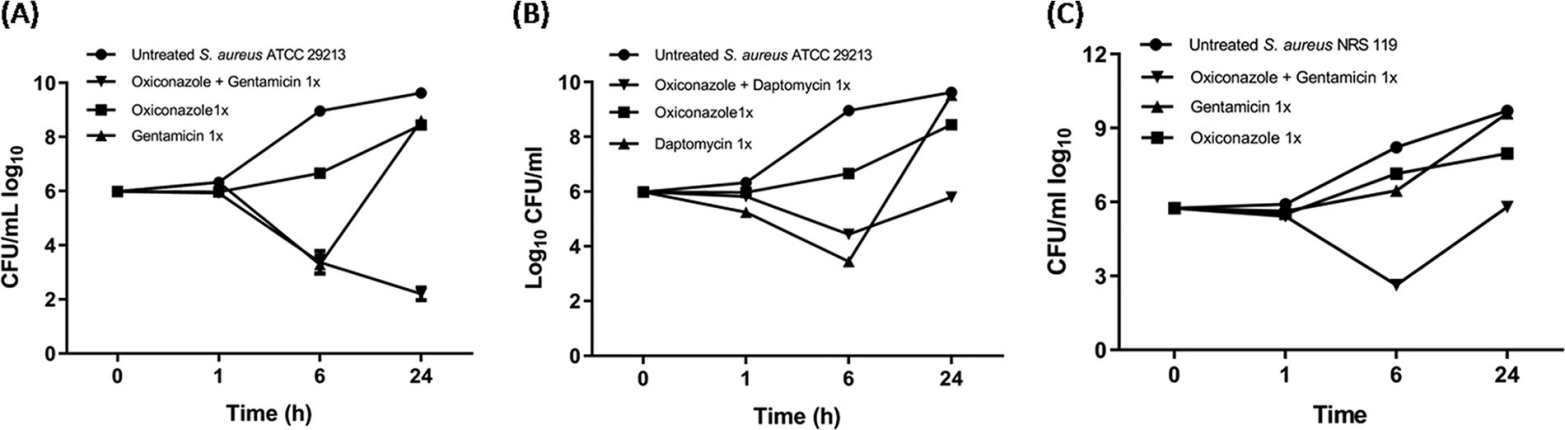
(A to C) Time-kill kinetics of oxiconazole in combination with (A) gentamicin and (B) daptomycin against S. aureus ATCC 29213 and (C) oxiconazole in combination with gentamicin against gentamicin-resistant S. aureus NRS 119. The error bars denote the standard error of the mean (SEM) gained from duplicate samples used for each antibiotic/compound examined in two independent experiments (*n* = 8). Time-kill kinetics of oxiconazole alone with comparator antibiotics and in combination with gentamicin and daptomycin against S. aureus ATCC 29213 was performed in the same set of experiments, and results are shown separately in Fig. 1 and this figure for clarity. CFU/mL Log_10_ values for the untreated control (S. aureus ATCC 29213) and the oxiconazole (1× MIC) treatment group are unchanged in this figure and Fig. 1.

Time-kill analysis was carried out using 1× MICs of oxiconazole alone and with daptomycin and gentamicin at their 1× MICs against S. aureus ATCC 29213 in order to further validate the observed synergy. The findings are shown in Fig. 2A and B. As can be seen in Fig. 2A, the combination of 1× MIC oxiconazole and gentamicin reduced the log_10_ CFU/mL by ~7.42 in 24 h, with no regrowth, which is much more potent than either drug alone. Similarly, the combination of 1× MIC oxiconazole and daptomycin reduced the log_10_ CFU/mL by ~3.83 compared to the untreated control in 24 h (Fig. 2B). Thus, the combinations of oxiconazole with daptomycin and gentamicin are much more potent than the individual drugs against S. aureus ATCC 29213 in reducing bacterial load.

To determine if oxiconazole is able to potentiate gentamicin against gentamicin-resistant S. aureus (GRSA) NRS119 (gentamicin MIC, 16 mg/L), its activity in combination was tested against GRSA NRS119, and the results are plotted in (Fig. 2C). As shown in the figure, oxiconazole activity with gentamicin was similar to its potent activity against S. aureus ATCC 29213 (Fig. 2C). In fact, the combination of oxiconazole and gentamicin reduced the log_10_ CFU/mL by ~3.9 in 24 h, which is more than the oxiconazole-alone (~1.7 log_10_ CFU/mL) and gentamicin-along (0.07 log_10_ CFU/mL) treatment groups, thus further exemplifying the potential of the combination, even against drug-resistant S. aureus. Since oxiconazole potentiates the activity of gentamicin against GRSA, it can prove to be a potential new antistaphylococcal therapeutic option, especially against infections caused by GRSA.

### Oxiconazole eradicates S. aureus ATCC 29213 preformed biofilm.

**FIG 3:**
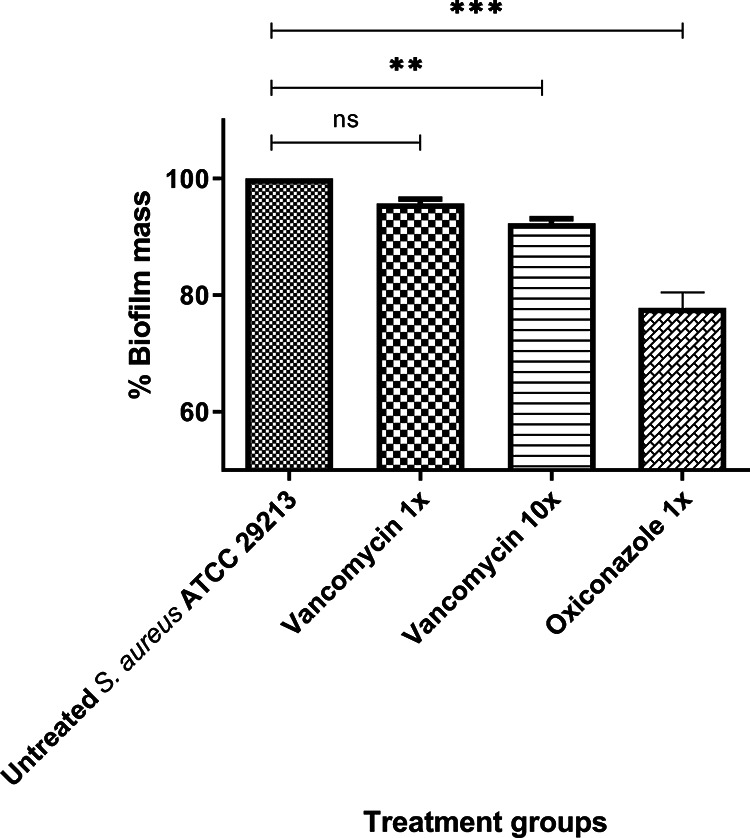
Biofilm inhibition by oxiconazole and comparator antibiotics against S. aureus ATCC 29213. The error bars symbolize the standard error of the mean (SEM) acquired from quadruplicate samples used for each antibiotic/compound examined in two independent experiments (*n* = 16). Statistical analysis was performed using Prism 8.0.2 software (GraphPad Software, La Jolla, CA, USA). Comparison between three or more groups was analyzed using one-way ANOVA with Dunnett’s multiple-comparison test. *P* values of <0.05 were considered significant. Adjusted *P* values between 0.01 and 0.05 *, between 0.01 and 0.001 (**), and between 0.001 and 0.0001 (***) are shown.

Many bacterial pathogens, including S. aureus, form biofilms as part of their growth cycle, and these shield bacteria from antibiotics and disinfectants, growing as a sessile microbial community lodged inside an amorphous slimy material (22). This often leads to persistent infections that are nonresponsive to antibiotics and cause of serious concern worldwide. Thus, oxiconazole’s ability to eradicate preformed S. aureus ATCC 29213 biofilms was assessed at 1× and 10× MIC of oxiconazole and vancomycin, and the results are shown in Fig. 3. As can be seen, treatment with 1× MIC of oxiconazole resulted in a significant decrease in biofilm mass compared to untreated biofilm (~22%, *P* < 0.005), while vancomycin at 10× MIC did not show significant activity (~7.7%, *P* < 0.5) (Fig. 3). Taking these data together, oxiconazole exhibits activity against bacteria in different physiological states from planktonic to biofilm, which is an important asset for a novel antimicrobial.

### Oxiconazole exhibits very long postantibiotic effect (PAE) against S. aureus ATCC 29213.

For any molecule under consideration, possessing a long PAE is a positive property, as it helps in minimizing the dosages required for therapeutic clearance (23). In this context, the PAE of oxiconazole was determined along with vancomycin and levofloxacin against S. aureus ATCC 29213, and the results are tabulated in Table 5. As shown, oxiconazole was found to exhibit a PAE of ~22 h at 10× MIC, which is much longer than that of vancomycin and levofloxacin (PAE of ~1 to 2 h at 10× MIC) against S. aureus (Table 5). Thus, oxiconazole exhibits concentration-dependent bactericidal activity with a very long PAE against S. aureus ATCC 29213, which is much better than vancomycin and levofloxacin.

**TABLE 5:** Postantibiotic effect of oxiconazole against S. aureus ATCC 29213

Antibiotic	Time for 1 log_10_ change (h)	PAE (h)
S. aureus ATCC 29213	2	0
Oxiconazole 1× MIC	~2	0
Oxiconazole 10× MIC	~24	~22
Levofloxacin 1× MIC	~3	~1
Levofloxacin 10× MIC	~4	~2
Vancomycin 1× MIC	~3	~1
Vancomycin 10× MIC	~3	~1

### S. aureus ATCC 29213 does not generate stable resistance to oxiconazole.

**FIG 4:**
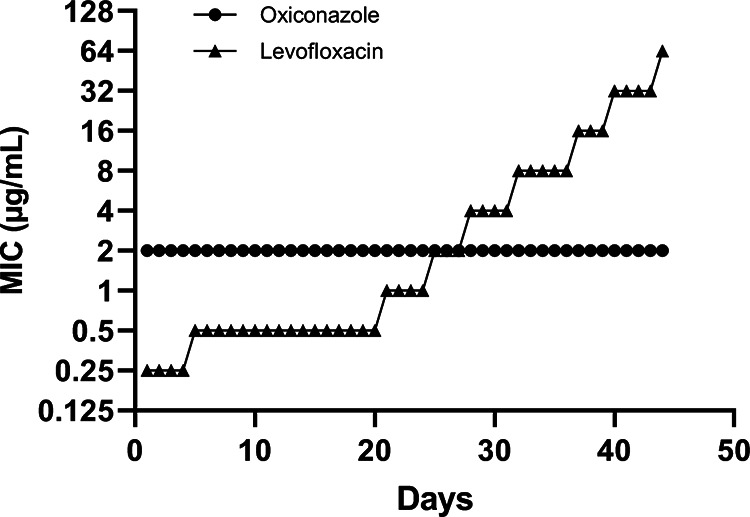
Absence of resistance to oxiconazole in S. aureus ATCC 29213. Triplicate culture samples of S. aureus ATCC 29213 were exposed to subinhibitory concentrations of oxiconazole and levofloxacin for a period of 40+ days, and the MIC was determined every 3rd day.

Since AMR is an ever-increasing worldwide problem, it is prudent to determine the propensity for resistance development in any potential antimicrobial drug candidate. Hence, the ability of S. aureus to generate resistance to oxiconazole and levofloxacin was assessed by continuous exposure to sublethal concentrations over a period of ~40 days; the results are plotted in Fig. 4. As shown, even after continuous exposure over 40 days in the presence of subinhibitory concentrations of oxiconazole, S. aureus did not generate resistance against oxiconazole. On the other hand, S. aureus generated stable resistance to levofloxacin, with its MIC increasing 256-fold against S. aureus over the same duration of serial passaging. The inability of S. aureus to generate stable resistance to oxiconazole augurs well for its clinical utilization (24).

### Oxiconazole is robustly active *in vivo* in a murine skin infection model against S. aureus ATCC 29213.

**FIG 5:**
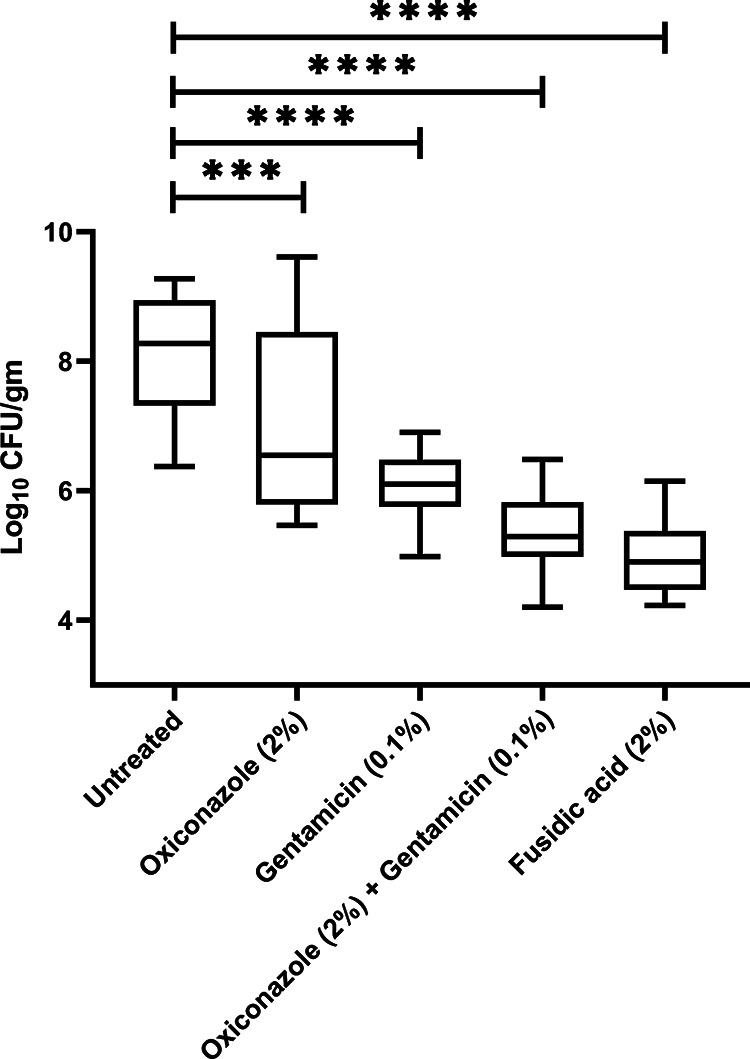
*In vivo* efficacy of oxiconazole alone and in combination with gentamicin against S. aureus ATCC 29213 in a murine skin infection model. Mice were treated twice daily with an 8 h interval; each treatment group comprised 4 mice caged individually. The error bars denote the standard deviations (SD) gained from triplicate samples used for each treatment group examined in two independent experiments (*n* = 24). Statistical analysis was performed using Prism 8.0.2 software (GraphPad Software, La Jolla, CA, USA). Comparison between three or more groups was analyzed using one-way ANOVA with Dunnett’s multiple-comparison test. *P* values of <0.05 were considered significant. ****, Adjusted *P* values between 0.0001 and 0.00001.

Since oxiconazole exhibited potent *in vitro* activity and synergized with gentamicin against both drug-susceptible S. aureus ATCC 29213 and GRSA NRS119, we tested the *in vivo* efficacy of oxiconazole and gentamicin combination in a murine superficial skin infection model, and the results are plotted in Fig. 5. As can be seen in Fig. 5, the combination of oxiconazole (2%) and gentamicin (0.1%) reduced the bacterial load in skin by ~2.77 log_10_ CFU/gm, which is significantly (*P* < 0.005) more than that of oxiconazole (~1.08 log_10_ CFU/gm) and gentamicin (~2.06 log_10_ CFU/gm) alone and is comparable to that of fusidic acid (~3.12 log_10_ CFU/gm). Taken together, these results indicate that the combination of Oxiconazole with gentamicin is potently active *in vivo* against S. aureus skin infections.

The unceasing emergence of AMR has negatively impacted morbidity and mortality worldwide, thus shining the spotlight on urgent discovery and clinical deployment of novel antimicrobials targeting WHO priority pathogens. In this study, we have explored in detail the antimicrobial potential of oxiconazole, alone and in combination with gentamicin, against drug-resistant S. aureus. Our study demonstrated that oxiconazole exhibited equipotent *in vitro* activity against drug-susceptible and well-characterized clinical multidrug-resistant strains of S. aureus and *Enterococcus* and is able to circumvent most prevalent resistance mechanisms present among these strains. When paired with an OM permeabilizer such as PMBN, oxiconazole expressed decent activity against GNB pathogens including carbapenem resistant A. baumannii (CRAB) BAA-1605, thus suggesting existence of a target in different bacterial species. Oxiconazole exhibits concentration-dependent bactericidal killing kinetics against S. aureus ATCC 29213 along with significant preformed biofilm eradication activity and a very long PAE of ~22 h. S. aureus was unable to generate stable resistance to oxiconazole, even after exposure for over 40 days, whereas under the same conditions, S. aureus generated resistance to levofloxacin. This lack of stable resistance generation by S. aureus is a positive indication for its clinical utilization (24). Furthermore, oxiconazole demonstrated ideal potential to synergize with gentamicin against drug-resistant S. aureus, including GRSA NRS119. Under *in vitro* conditions against both drug-susceptible S. aureus ATCC 29213 and GRSA NRS119, the combination of 1× MIC of oxiconazole and gentamicin comprehensively inhibited S. aureus culture with no regrowth. Similarly, under *in vivo* conditions, the combination of oxiconazole (2%) and gentamicin (0.1%) more significantly reduced the bacterial load in a skin infection model than did either drug alone and is comparable to the reduction caused by fusidic acid (2%), a drug typically utilized for the treatment of staphylococcal skin infections.

The combination of oxiconazole with gentamicin is very fortuitous since both drugs are extensively utilized for topical application, with gentamicin being selectively utilized in skin and skin structure bacterial infections (25). Oxiconazole nitrate is an antifungal imidazole derivative commercially available as 1% oxiconazole nitrate cream and clinically utilized for topical treatment of tinea pedis, tinea cruris, tinea corporis, and tinea (pityriasis) versicolor caused by dermophytes such as Trichophyton rubrum, Trichophyton mentagrophytes, Epidermophyton floccosum, and Malassezia furfur (26). It was jointly developed by F. Hoffmann-LaRoche and Siegfried AG (Basel, Switzerland) and became available in United States in 1989 as a once-daily treatment (26–28). Oxiconazole possesses broad-spectrum *in vitro* antifungal activity against Candida albicans, Microsporum audouinii, Microsporum canis, Microsporum gypseum, Trichophyton tonsurans, Trichophyton violaceum, etc. (28–31). The antifungal activity is reported to be primarily derived from inhibition of ergosterol biosynthesis, which is critical to fungal cell membrane integrity, by destabilization of the fungal cytochrome P450 51 enzyme (also known as lanosterol 14-alpha demethylase), leading to cell lysis (28). Subinhibitory concentrations of oxiconazole are reported to inhibit DNA synthesis, suppress intracellular concentrations of ATP, and slightly decrease RNA, protein, and carbohydrate synthesis (28). Due to variable responses obtained in *in vivo* studies following oral administration, oxiconazole was developed as a topical antifungal agent with negligible systemic absorption through skin (26, 28, 29). However, there is rapid absorption of the drug into the stratum corneum of epidermis, with maximum concentrations being attained within 100 min of its application on skin. Oxiconazole also exhibits deep skin penetration and long retention time, where fungicidal concentrations are maintained in epidermis, upper corium, and deeper corium for at least 5 h after its application, thus making it a useful topical agent (26, 32). Gentamicin is commercially available as 0.1% gentamicin sulfate in a cream or ointment base, and no significant systemic absorption at levels associated with aminoglycoside toxicity has been observed with its topical administration on infected areas in infants and adults at the directed dose (33). However, many studies have reported that topical monotherapy with gentamicin may lead to dissemination of resistant strains (34–37). Also, it has been observed that topical antibiotic use (33) promotes the overgrowth of other nonsusceptible organisms that primarily include fungi. Oxiconazole proves to be an ideal candidate for repurposing for combination therapy with gentamicin owing to its extremely low propensity for resistance generation in S. aureus, activity against MDR strains, broad antifungal efficacy, and excellent safety and tolerability profile (25). Thus, oxiconazole’s activity in combination with gentamicin offers a promising path ahead for its clinical utilization as a novel therapeutic targeting mixed fungal and bacterial superficial skin infections.

## MATERIALS AND METHODS

### Growth media and reagents.

All the bacterial strains used in the study were routinely cultivated in culture media suited to them, which included Mueller-Hinton cation-supplemented broth II (MHBII), Mueller-Hinton agar (MHA), and tryptic soy broth (TSB) purchased from Becton, Dickinson and Company (BD; USA). The chemicals and antibiotics used were procured from Sigma-Aldrich (USA). The Prestwick chemical library, containing 1,520 approved compounds, was available in-house, with all compounds resuspended in 100% dimethyl sulfoxide (DMSO) at a 10-mM concentration. RPMI and fetal bovine serum (FBS) used in eukaryotic cell culture maintenance were purchased from MP Biomedicals and Gibco (USA origin), respectively. All methods were performed in accordance with the relevant guidelines and regulations.

### Bacterial strains and eukaryotic cell lines.

Oxiconazole was screened against a bacterial pathogen panel consisting of Escherichia coli ATCC 25922, Staphylococcus aureus ATCC 29213, Klebsiella pneumoniae BAA-1705, Acinetobacter baumannii BAA-1605, Pseudomonas aeruginosa ATCC 27853, and *Enterococcus* spp. The panel was further expanded to include drug-resistant clinical S. aureus and *Enterococcus* strains, including those resistant to vancomycin and other clinically utilized antibiotics. These strains were procured from the Biodefense and Emerging Infections Research Resources Repository/Network on Antimicrobial Resistance in Staphylococcus aureus/American Type Culture Collection (BEI/NARSA/ATCC, USA) and routinely cultivated on MHA, MHBII, and TSB. Vero (ATCC CCL-81) cells were procured from ATCC (USA) and routinely maintained in RPMI medium supplemented with 10% FBS.

### Antibiotic susceptibility testing.

Antibiotic susceptibility testing was conducted according to CLSI guidelines using the broth microdilution assay (38). Stock solutions of test compounds (10 mg/mL) were prepared in DMSO. Bacterial cultures were inoculated in MHBII, and the optical density (OD) was measured at 600 nm, followed by dilution to achieve ~10^6^ CFU/mL. The compounds were tested from 64 to 0.5 mg/L in a 2-fold serially diluted fashion with 2.5 μL of each concentration added to a well of a 96-well round-bottom microtiter plate. Later, 97.5 μL of bacterial suspension was added to each well containing either test compound or appropriate controls. The plates were incubated at 37°C for 18 to 24 h, following which the MIC was determined. The MIC is defined as the lowest concentration of the compound at which there is an absence of visible growth. For each test compound, MIC determinations were carried out independently three times using duplicate samples.

### Screening of the Prestwick Chemical Library against clinically relevant human bacterial pathogens.

The Prestwick chemical library was screened to identify hits active against clinically relevant human bacterial pathogens as specified above. The compounds (2 μL) were added to 96-well round-bottom microtiter plate (Corning) followed by 98 μL of bacterial inoculum, resulting in a final drug concentration of 50 μM in the primary screen. Wells containing only cells (positive control) or medium were included on each assay plate, and the plate was incubated at 37°C for 18 to 24 h to determine MIC. After the initial hit determination, the hit compounds were serially diluted to determine the MIC of specific compounds.

### PMBN assay.

The MIC of oxiconazole against Escherichia coli ATCC 25922 and Acinetobacter baumannii BAA-1605 was determined in the presence of 10 mg/L polymyxin B nonapeptide (PMBN) in culture broth according to the method utilized for MIC determination (38, 39).

### Bacterial time-kill kinetics with oxiconazole.

The presence or absence of bactericidal activity was assessed using a time-kill kinetics method as described (40). Briefly, S. aureus ATCC 29213 was diluted to ~10^6^ CFU/mL in MHBII and treated with 1× and 10× MIC of oxiconazole and vancomycin and incubated at 37°C with shaking for 24 h. Then, 0.1-mL samples were collected at 0, 1, 6, and 24 h, serially diluted in MHBII, and plated on TSA followed by incubation at 37°C for 18 to 20 h. The kill curves were constructed by counting the colonies from plates and plotting the CFU/mL of surviving bacteria at each time point in the presence and absence of compound. Each experiment was repeated three times in duplicate, and the mean data were plotted.

### Drug interaction of oxiconazole with FDA-approved drugs.

The interaction of oxiconazole with FDA-approved drugs, namely, vancomycin, levofloxacin, ceftazidime, linezolid, daptomycin, meropenem, minocycline, rifampicin, and gentamicin was tested using the checkerboard method (41). Serial 2-fold dilutions of each drug were freshly prepared prior to testing. Oxiconazole was 2-fold-diluted along the ordinate, while the antibiotics were serially diluted along the abscissa in a 96-well microtiter plate. Then, 95 μL of ~10^5^ CFU/mL S. aureus ATCC 29213 was added to each well, and the plates were incubated at 37°C for 24 h. After incubation, ΣFICs (fractional inhibitory concentrations) were calculated as follows: ΣFIC = FIC A + FIC B, where FIC A is the MIC of drug A in combination/MIC of drug A alone and FIC B is the MIC of drug B in combination/MIC of drug B alone. The combination is considered synergistic when the ΣFIC is ≤0.5, indifferent when the ΣFIC is >0.5 to 4, and antagonistic when the ΣFIC is >4 (41).

The synergism was further validated via time-kill kinetics assays performed as described above. S. aureus ATCC 29213 and gentamicin-resistant S. aureus NRS119 were treated with 1× MIC of both oxiconazole and synergistic drugs alone and in combination. Then, 0.1-mL samples were collected at 0, 1, 6, and 24 h, serially diluted in MHBII, and plated on TSA, followed by incubation at 37°C for 18 to 20 h. The kill curves were constructed by counting colonies from plates and plotting the CFU/mL of surviving bacteria at each time point in the presence and absence of compound. Each experiment was repeated three times in duplicate, and the mean data were plotted.

### Determination of oxiconazole’s activity in eradicating preformed S. aureus biofilm.

The determination of oxiconazole’s antibiofilm activity was performed as described earlier (40, 42). Briefly, S. aureus ATCC 29213 was grown overnight in TSB containing 1% glucose with shaking (180 RPM) at 37°C. The overnight culture was diluted in fresh TSB broth containing 1% glucose (1:100), and 0.2 mL of this freshly diluted culture was transferred into 96-well polystyrene flat-bottom plates, covered with an adhesive foil lid for maintaining low oxygen, and incubated under static conditions for 48 h at 37°C. After incubation, medium was decanted, and the plates were rinsed gently 3 times with the 1× phosphate-buffered saline (PBS; pH 7.4) to remove the planktonic bacteria. The plates were refilled with TSB containing oxiconazole at 1× MIC and vancomycin at 1×and 10× MIC and incubated for 24 h at 37°C. After drug treatment, the medium was decanted and washed 3 times with 1× PBS (pH 7.4), and the biofilm was fixed by incubating the plate at 60°C for 1 h. After fixing, the biofilm was stained by 0.06% crystal violet for 10 min, rinsed with PBS, and dried at room temperature. For quantification of biofilm, bound crystal violet was eluted by 30% acetic acid (0.2 mL in each well). Absorbance was taken on a microtiter plate reader at 600 nm for biofilm quantification.

### Determination of the postantibiotic effect (PAE) of oxiconazole.

To determine the PAE of oxiconazole, overnight culture of S. aureus ATCC 29213 was diluted in MHBII at ~10^5^ CFU/mL and exposed to 1× and 10× MIC of vancomycin, levofloxacin, and oxiconazole and incubated at 37°C for 1 h. After the 1-h treatment, culture was centrifuged and washed 2 times with prewarmed MHBII to remove any traces of antibiotics. Finally, cells were resuspended in drug-free MHBII and incubated further at 37°C. Samples were taken after every 1 h, serially diluted, and plated on TSA for enumeration of CFU. The PAE was calculated as PAE = *T* – *C*, where *T* is the time required for a 1 log_10_ increase in CFU compared to the CFU observed immediately after the removal of drug, and *C* is the time difference in a similarly treated drug-free control (23).

### Determination of induced resistance generation in S. aureus against oxiconazole.

The propensity of bacteria to generate resistance can be determined using serial exposure of organisms to subinhibitory concentrations of antimicrobial agents (43). Hence, the potential emergence of resistance in S. aureus ATCC 29213 against oxiconazole was assessed as described before (40). For this purpose, S. aureus ATCC 29213 was subjected to serial passages in the presence of subinhibitory concentrations of oxiconazole and levofloxacin for over 30 days, and changes in MIC values were monitored every third passage. The change in MIC is plotted against time under culture.

### Murine skin infection model with oxiconazole alone and in combination with gentamicin against S. aureus ATCC 29213.

The superficial skin infection experiments were performed as described previously (44). Male, Swiss mice (4 to 6 weeks old) weighing between 24 and 26 g were used for all experiments. The mice were caged singly in individually vented cages (IVC) under aseptic conditions for mitigating the risk of cross-contamination during the course of the experiment. The experiment was initiated by anesthetizing the mice by injecting a 100-μL mixture of ketamine and xylazine intraperitoneally (i.p.) at 40 mg/kg and 8 mg/kg of body weight, respectively, in distilled water. The fur was then removed from the mice with the aid of a depilatory cream, and the area was cleaned with sterile distilled water. An area of ~2 × 2 cm^2^ was scratched until the skin became visibly damaged and was characterized by reddening and glistening without any bleeding. A 10-μL droplet containing ~10^7^ CFU/mL S. aureus ATCC 29213 was then placed over the reddened area to initiate bacterial infection. In each experiment, a group of untreated mice were sacrificed 4 h postinfection to determine the infectious dose, and topical test treatments were started at the same time. Next, the treatment dose was applied at 16 h after the first treatment, and henceforth, the drug regimen (25 to 30 mg) comprised twice daily application (in morning and evening, with an 8 h interval) for 4 days. The treatment groups comprised untreated mice and mice treated with 2% fusidic acid (positive control), 2% oxiconazole, 0.1% gentamicin, and 2% oxiconazole + 0.1% gentamicin. All treatment groups were sacrificed 18 h after the last topical treatment to avoid carryover effects. After sacrifice, the ~2 × 2-cm^2^ wounded skin areas were excised aseptically and homogenized with 500 μL phosphate-buffered saline (1× PBS) in 2-mL MP tissue-grinding lysing matrix F tubes using an MP FastPrep-24 device set at 4.0 M/S for 30 s (3 cycles). After homogenization, suitable dilutions were plated on TSA plates and incubated at 37°C overnight for determining the number of surviving bacteria in each treatment group. Experiments were repeated thrice, the mean log_10_ CFU/gm was calculated for each experiment, and the average mean and standard error of the mean (SEM) of all three experiments were plotted.

### Ethics approval.

The use of mice for infectious studies (IAEC/2019/2/renew-1/dated-22.06.2020) was approved by the Institutional Animal Ethics Committee at CSIR-CDRI, Lucknow, India.

### Statistical analyses.

Statistical analysis was performed using Prism 8.0.2 software (GraphPad Software, La Jolla, CA, USA). Comparison between three or more groups was analyzed using one-way analysis of variance (ANOVA) with Dunnett’s multiple-comparison test. *P* values of <0.05 were considered significant.
